# *UCA1* copy number gain in serum cfDNA predicts next-generation antiandrogen resistance in metastatic castration-resistant prostate cancer

**DOI:** 10.1038/s41598-026-56189-y

**Published:** 2026-06-21

**Authors:** Anita Csizmarik, Áron Soós, Melinda Váradi, Balázs Magyar, Martin Puhr, Ákos Nagy, Balázs Győrffy, Gergő Holló, Gero Kramer, Mulham Al-Nader, Osama Mahmoud, Boris Hadaschik, Péter Nyirády, Tibor Szarvas

**Affiliations:** 1 Department of Urology, Semmelweis University, Üllői street 78/b, Budapest, H-1082 Hungary; 2 Department of Urology, Medical University of Innsbruck, Innsbruck, Austria; 3 HCEMM-SE Molecular Oncohematology Research Group, Department of Pathology, Experimental Cancer Research, Semmelweis University, Budapest, Hungary; 4 Department of Bioinformatics, Semmelweis University, Budapest, Hungary; 5 Department of Biophysics, University of Pecs, Pecs, Hungary; 6 HUN-REN Research Centre for Natural Sciences, Institute of Molecular Life Sciences, Budapes, Hungary; 7 Department of Urology, Medical University of Vienna, Vienna, Austria; 8 Department of Urology, University Hospital Essen, University of Duisburg-Essen, Hufelandstr. 55, Essen, D-45147 Germany; 9 Department of Urology, South Valley University, Qena, Egypt

**Keywords:** Cell-free DNA (cfDNA), Prostate cancer, Biomarker, *UCA1*, Androgen receptor, Biomarkers, Cancer, Oncology, Urology

## Abstract

Enzalutamide (ENZA) is a second-generation antiandrogen therapy that provides overall survival (OS) benefit for prostate cancer (PC) patients. However, many patients have baseline or develop resistance to ENZA. Currently, no biomarkers are used in clinical decision making to predict the efficacy of ENZA therapy. We aimed to identify cell-free DNA (cfDNA) biomarkers for metastatic castration-resistant prostate cancer (mCRPC) predictive to ENZA response. Comparative whole-exome and transcriptome sequencing was performed on three ENZA-resistant and parental ENZA-sensitive PC cell line pairs. Bioinformatic analysis identified *UCA1*,* ORM1*, and androgen receptor (AR) as candidate markers for serum cfDNA testing. Digital droplet PCR was used to analyze *UCA1*,* ORM1* and *AR* in cfDNA from serum samples of mCRPC patients who underwent ENZA (*n* = 20), abiraterone (ABI; *n* = 20) and 40 docetaxel (DOC; *n* = 40) treatment. *UCA1* copy number gain was significantly associated with reduced OS in ENZA-treated patients (*p* = 0.030). *AR* gain correlated with poorer OS in ABI-treated patients (*p* = 0.039), whereas no association was observed in DOC-treated patients. Normal *AR* and *UCA1* levels were associated with significantly better OS in ENZA and ABI-treated patients. These findings suggest that *UCA1* gain may predict reduced efficacy of ENZA treatment, while *AR* gain indicates decreased effectiveness of ABI but not ENZA and DOC therapies. These results may help support therapeutic decisions in the clinical settings.

**Supplementary Information:**

The online version contains supplementary material available at https://doi.org/10.1038/s41598-026-56189-y.

## Introduction

In recent years, the treatment landscape for castration-resistant prostate cancer (CRPC) has undergone significant changes. Many treatments have become available, including chemotherapy, next-generation antiandrogen therapy, immunotherapy, PARP inhibitors, and radiopharmaceuticals. Enzalutamide (ENZA) is a next-generation antiandrogen used in metastatic CRPC (mCRPC) in the post-chemotherapy setting^1^. Later, based on the PREVAIL study, ENZA became available for chemotherapy-naïve and non-metastatic CRPC patients^2,3^. More recently, ENZA has been applied in treatment intensification in hormone-sensitive prostate cancer patients and is under investigation in earlier settings^4,5^.

Although ENZA provides an overall survival (OS) benefit, baseline or acquired resistance limits efficacy and causes differences in outcomes. Several mechanisms may underlie ENZA resistance, including alterations in androgen receptor (AR), such as AR amplification, and expression of AR splice variants. DNA- and Wnt-pathway alterations and *TP53* or *RB1* loss may also be involved^6^. Accordingly, some potential markers of therapy resistance, including glucocorticoid receptor or PD-L1 overexpression, and elevated neuroendocrine markers (chromogranin A, neuron-specific enolase), have been identified^6^. Using a systematic proteomics approach, our group identified ALCAM and FSCN1 as potential serum markers for ENZA and ABI treatment of mCRPC^7,8^. However, none of these biomarkers are currently used in routine clinical practice.

As in mCRPC tumor tissue is difficult to access, blood-based cell-free DNA (cfDNA) analysis represents an easy to access and non-invasive technique to support therapeutic decisions^9,10^. CfDNA, a fraction of circulating nucleic acids, can be detected in serum or plasma^11^. Several studies highlight its utility in prostate cancer (PC). One of the largest studies with 3,129 mCRPC cases showed that 94% of patients had detectable plasma cfDNA, of which 42% had *AR* alterations and 8.8% *BRCA1/2* mutations^12^. Moreover, these abnormalities correlated with alterations isolated from matched tumor tissue samples^12^. Further cfDNA studies have shown that *AR* gain in cfDNA associated with shorter OS in ENZA and ABI treated patients^13,14^. However, not only AR alterations, but also mutations in the DNA damage response pathway or PTEN-PI3K-AKT pathway aberrations detected in cfDNA may be associated with resistance to therapy^15,16^.

In this study, we aimed to identify cfDNA biomarkers in serum samples from patients with ENZA-treated mCRPC. For this, we performed whole exome and transcriptome sequencing on three ENZA-sensitive and ENZA-resistant PC cell lines and compared the sequencing results; two genes were selected for amplification analysis. Moreover, we selected *AR* because its copy number (CN) gain is known to be associated with a poor next-generation antiandrogen response. Digital droplet PCR (ddPCR) was used to analyze *Urothelial cancer associated 1 (UCA1)*,* Orosomucoid 1 (ORM1)* and *AR* in cfDNA from serum samples of mCRPC patients. As we found that *UCA1* CN gain is associated with poor survival in ENZA-treated patients, we extended its analysis to abiraterone (ABI) and docetaxel (DOC)-treated mCRPC patients.

## Results

### Identification of genes for serum cfDNA analysis

**Fig. 1:**
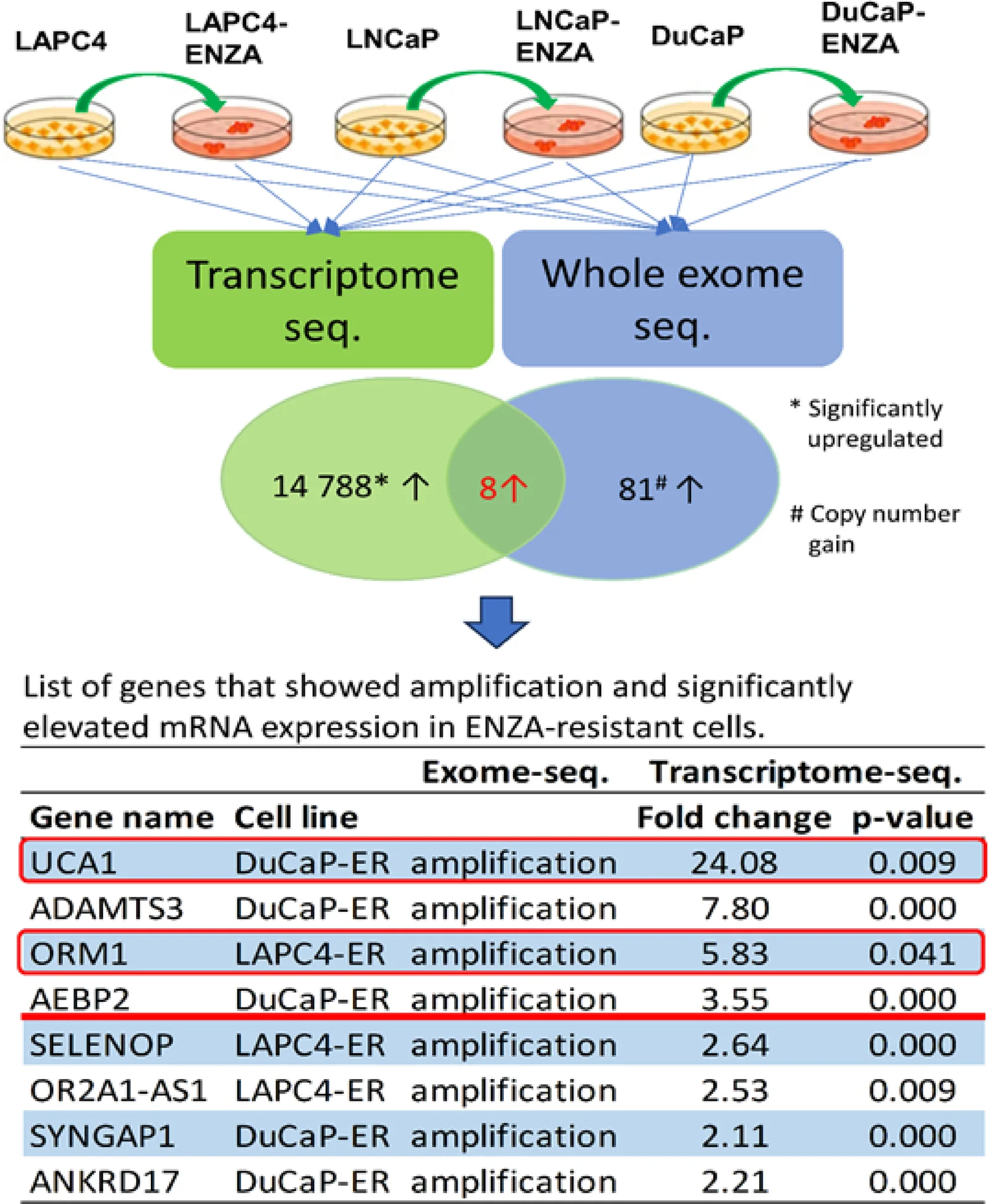
Identification of genes for serum cfDNA analysis. Genes with CN gain and significantly elevated mRNA levels in ENZA resistant cell lines were selected.

Whole exome sequencing identified 81 genes that were amplified, and transcriptome sequencing revealed 14,788 genes with significantly elevated mRNA expression in ENZA-resistant cells. We identified eight genes that were amplified and also had significantly elevated mRNA expression levels (Fig. 1). *UCA1* was selected for serum cfDNA analysis because it showed amplification and the highest fold-change (24x) by transcriptome sequencing. *ORM1* was selected because of its amplification and 5.8 × increased gene expression in ENZA-resistant compared to ENZA-sensitive parental cell lines. Moreover, we selected *AR* because its copy CN gain is known to be associated with a poor anti-androgen response.

### Amplification analysis of selected genes in serum cfDNA samples

#### Patients’ characteristics

In the ENZA cohort, the median age was 73 years (range: 61–87), and the median baseline PSA value was 46.5 ng/ml. The primary endpoint of the analysis was overall survival (OS). Fourteen ENZA-treated patients died during the median follow-up period of 27 months (Table 1).

The median age of the ABI cohort was 69 years (range: 50–84) and the median PSA value was 32.9 ng/ml. Nine of the 20 ABI-treated patients died during the median follow-up period of 25 months (Table 1). None of the ABI-treated patients received ENZA treatment before or after ABI.

In the DOC cohort, the median patient age was 70 years (range: 49–87), and the median PSA value was 77.1 ng/ml. In this cohort, 26 patients died during the median follow-up period of 24 months (Table 1).

**Table 1:** Patients’ characteristics.

Parameters	ENZA cohort	ABI cohort	DOC cohort
Total Nr. of patients	20	20	40
Age years (range)	73 (61–87)	69 (50–84)	70.3 (49–87)
PSA (ng/ml)	46.5 (1.4–4341.9)	32.9 (0.1–941.0)	77.11 (1.0–6115.4)
ECOG PS (%) 0	14	15	17
1	5	4	19
Unknown	1	1	4
Metastases			
Bone	17	19	32
LN (> 2 cm) or/and visceral met.	9	10	20
Primary local therapy	12	8	8
PSA response (%) >30	13	14	26
>50	12	12	20
>90	8	6	13
Nr. of patients died (censored)	14 (6)	9 (11)	26 (14)
OS median (range)(months)	26.6 (0.8–61.0)	25.3 (3.1–70.3)	23.9 (2.0–61.2)

#### Correlations of clinicopathological parameters with gene amplification

We found no significant correlations between the assessed gene amplification and ENZA- or ABI-treated patient age, baseline PSA level, ECOG status, presence of metastases, primary local therapy, or PSA response (Supplementary Tables 1,2). However, when the ENZA and ABI cohorts were merged, we found significantly higher baseline PSA levels in patient samples with *UCA1* gene amplification (Supplementary Table 3). In the DOC cohort, we found significantly higher PSA levels in patients without *UCA1 CN gain* (Supplementary Table 4).

#### Survival analyses

**Fig. 2:**
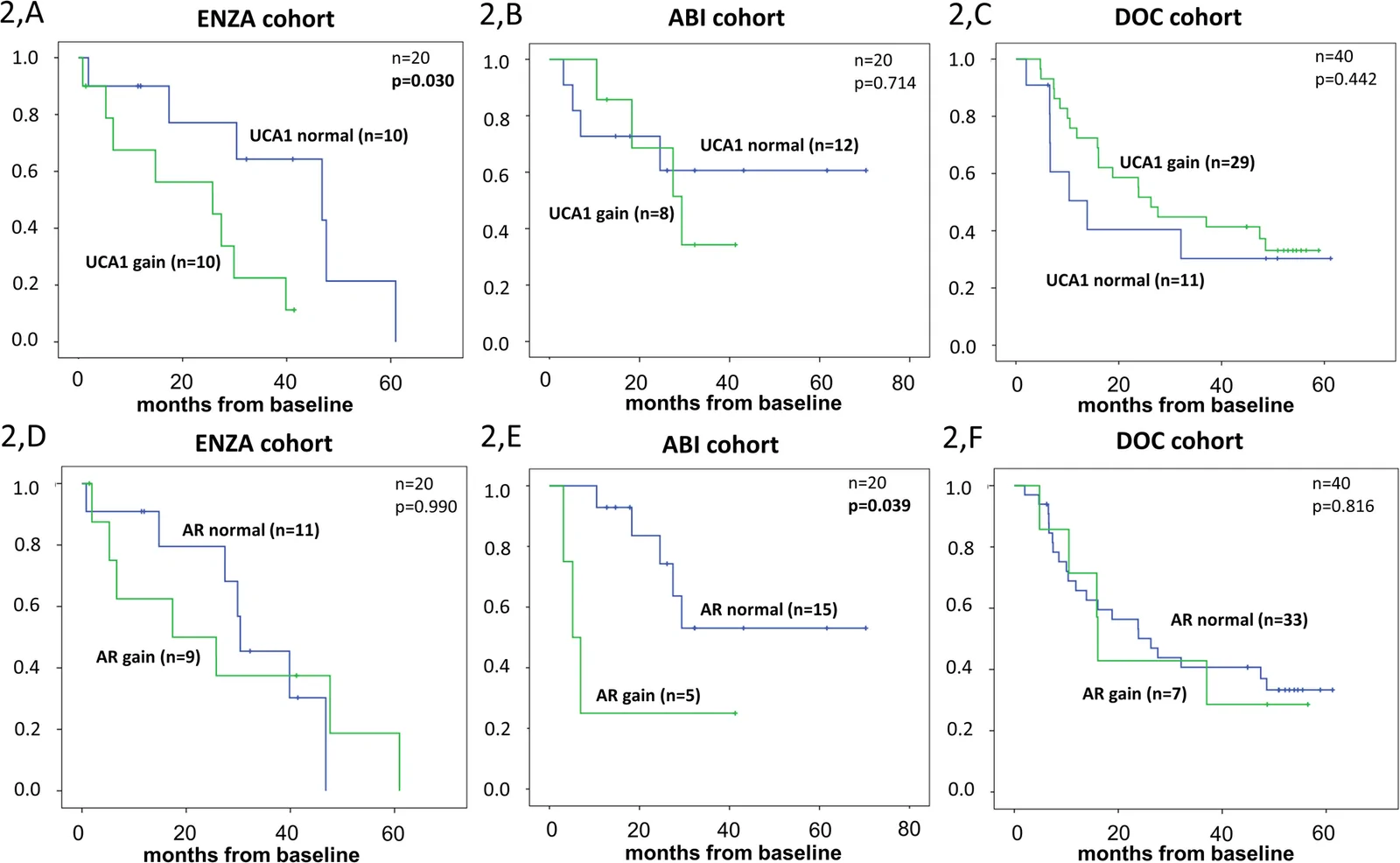
Kaplan-Meier analysis of *UCA1* and *AR* in ENZA, ABI, and DOC-treated cohort. Significant values are indicated in bold.

In the ENZA cohort, any PSA decrease, as well as at least a 30% and 50% PSA decrease, were significantly associated with favourable survival (*p* = 0.012, *p* = 0.015, and *p* = 0.015, respectively). *UCA1* gene amplification was significantly associated with shorter OS (*p* = 0.044) (Table 2.). Moreover, multivariable analyses with ECOG, PSA and visceral metastases revealed *UCA1* gain as an independent predictor of OS (Supplementary Tables 5,6). However, this analysis should be handled with caution, as the limited sample size restricts the statistical power and generalizability of the findings. The Kaplan-Meier OS curve revealed that *UCA1* CN gain was significantly associated with shorter OS (*p* = 0.030) (Fig. 2A).

In the ABI cohort, *AR* gene amplification tended to be associated with shorter OS; however, this correlation was not significant (*p* = 0.060) (Table 2.). However, Kaplan-Meier analyses revealed that *AR* gene amplification was significantly associated with worse OS (*p* = 0.039) (Fig. 2E). As *ORM1* CN gain was not associated with OS in the ENZA and ABI cohorts, it was not further analyzed in the DOC cohort.

**Fig. 3:**
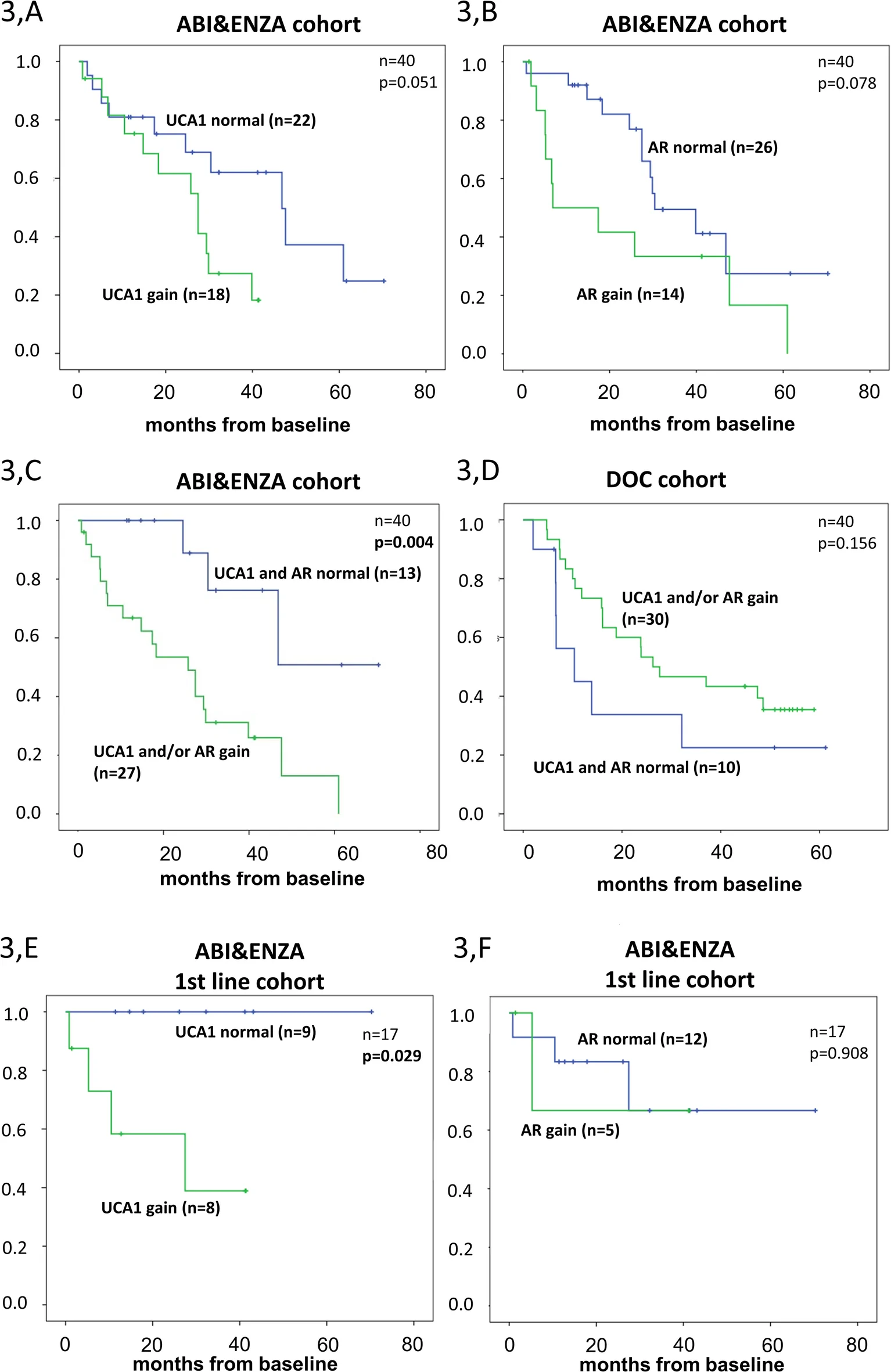
Kaplan-Meier analysis of *AR* and *UCA1* amplification in ENZA&ABI, DOC and ENZA&ABI 1 st line treated cohort. Significant values are indicated in bold.

In the DOC cohort, we found no significant correlations between either the assessed clinicopathological data or assessed gene amplifications (*AR* and/or *UCA1*) and OS (Table 2.; Fig. 2C,F, Fig. 3D).

In the merged ENZA&ABI cohort, Kaplan-Meier OS curves revealed a borderline association between *UCA1* CN gain and shorter OS (*P* = 0.051) (Fig. 3A). Furthermore, when we analyzed *UCA1* amplification in the first-line ENZA-ABI treated cohort, we obtained an even stronger significance value (*p* = 0.029) (Fig. 3E).

Moreover, we combined *UCA1* and *AR* gene amplifications for OS analysis and found that in the *UCA1* and *AR* non-amplified groups, ENZA/ABI-treated men had significantly better OS than those who had either *AR*, *UCA1* or both gene amplifications (*p* = 0.004) (Fig. 3C).

#### Gene set enrichment analysis (GSEA) of ENZA-resistant vs. sensitive cells

Results from the GSEA are provided in the Supplementary Materials.

#### Gene expression analysis of *UCA1* with selected signaling pathways

To investigate the molecular changes with *UCA1* copy number alterations we used publicity available DNA and RNA prostate cancer datasets. To assess the distribution of copy number gains and losses across genes in relation to *UCA1* status we performed genome-wide CNV analyses (Supplementary Tables 7, 8, 9) We did not find significant correlations *UCA1* gene expression and AR or PI3K/AKT pathways. However, we find that the expression of the tumor suppressor *CLCA4* (and CLCA2 as well) is significantly reduced in the presence of *UCA1* gain (*p* = 2.7 × 10 − 9, OR = 5.9), which suppresses the PI3K/AKT pathway. Moreover, we find that *AKT2* loss co-occurred significantly with *UCA1* gain (*p* = 0.0079, OR = 24.27), whereas *AR* loss was significantly less frequent in cases with *UCA1* gain (*p* = 8.0 × 10⁻⁵, OR = 0.034).

**Table 2:** Cox regression analysis of overall survival by clinical and molecular variables in ENZA, ABI, and DOC treatment groups. Significant values are in bold.

Variables		n	ENZA			n	ABI			n	DOC		
			Overall survival				Overall survival				Overall survival		
			HR	95% CI	p		HR	95% CI	p		HR	95% CI	p
Age	>72 y	13	1.255	0.384 - 4.099	0.707	8	3.453	0.821 - 14.526	0.091	15	1.200	0.543 - 2.652	0.652
ECOG	>1	5	1.852	0.594 - 5.778	0.288	4	1.747	0.345 - 8.848	0.500	9	3.636	1.708 - 7.743	**<0.001**
Visceral mets.	pos.	3	0.619	0.078 - 4.906	0.650	4	2.090	0.495 - 8.814	0.316	9	1.130	0.451 - 2.829	0.795
LN mets.	pos.	6	1.184	0.310 - 4.522	0.805	6	0.589	0.118 - 2.951	0.520	14	0.754	0.335 - 1.697	0.495
Bone mets.	pos.	17	27.770	0.038 - 20308.32	0.323	19	0.518	0.061 - 4.360	0.545	32	2.435	0.729 - 8.137	0.148
Primary treatment	pos.	12	0.278	0.075 - 1.034	0.056	8	0.603	0.121 - 3.005	0.537	8	0.527	0.180 - 1.540	0.242
Primary RPE	pos.	10	0.711	0.236 - 2.148	0.546	5	0.405	0.050 - 3.298	0.398	3	0.039	0.000 - 6.337	0.212
Primary RT	pos.	5	0.648	0.197 - 2.126	0.474	3	1.370	0.166 - 11.281	0.770	6	0.884	0.304 - 2.573	0.821
PSA median	*	10	2.298	0.737 - 7.168	0.152	10	3.963	0.786 - 19.973	0.095	20	1.341	0.619 - 2.903	0.457
PSA response	present	15	0.189	0.052 - 0.691	**0.012**	14	0.607	0.145 - 2.545	0.495	29	0.529	0.178 - 1.578	0.254
PSA response	>30%	13	0.224	0.067 - 0.744	**0.015**	14	0.607	0.145 - 2.545	0.495	26	0.409	0.162 - 1.033	0.059
PSA response	>50%	12	0.215	0.062 - 0.745	**0.015**	12	0.450	0.112 - 1.812	0.261	20	0.644	0.273 - 1.519	0.314
PSA response	>90%	8	0.401	0.108 - 1.487	0.172	6	0.222	0.027 - 1.810	0.160	13	0.548	0.212 - 1.415	0.214
AR gain	yes	9	0.993	0.309 - 3.190	0.990	5	4.141	0.963 - 17.809	0.056	7	1.123	0.423 - 2.982	0.816
UCA1 gain	yes	10	3.971	1.039 - 15.184	**0.044**	8	1.295	0.323 - 5.197	0.715	29	0.712	0.299 - 1.699	0.444
ORM1 gain	yes	15	0.471	0.131 - 1.695	0.250	14	1.160	0.274 - 4.915	0.840	–	–	–	–

Significant values are in bold

## Discussion

In the present study, we performed exome and transcriptome sequencing of three ENZA-resistant vs. ENZA-sensitive PC cell lines. Using this method, we selected *UCA1* and *ORM1* for amplification analysis, which were measured in serum cfDNA samples of mCRPC patients. Our ddPCR analysis revealed that *UCA1* amplification is significantly associated with worse OS in ENZA-treated patients, whereas in the ABI cohort, *AR* gain was significantly associated with poor OS. Moreover, those ENZA and ABI-treated patients with normal AR and *UCA1* gene expression had significantly better OS. Furthermore, our GSEA revealed pathways significantly downregulated in ENZA-resistant cells, such as protein synthesis, immune response, and metabolic processing, whereas upregulated pathways included cell adhesion, GnRH secretion, and cholesterol metabolism in mediating resistance.

*UCA1* is an oncogenic long non-coding RNA and a member of the human endogenous retrovirus H family^17^. *UCA1* was first identified in bladder cancer cell lines^18^, and later linked to colorectal, lung, gastric, breast, and ovarian cancers^17^. Most studies reported overexpression of *UCA1* in tumor vs. normal tissues, and elevated *UCA1* levels were linked to advanced tumor stage, positive lymph node status, larger tumor size, and poor OS^19,20^. In PC, Zhang et al. found that *UCA1* was significantly overexpressed in tumor tissue and cell lines. *UCA1* overexpression correlated with a high Gleason score, advanced tumor stage, and poor OS. Moreover, *UCA1* silencing suppressed PC cell migration, proliferation, and invasion^21^. Another study reported high *UCA1* expression associated with higher Gleason score, and knockdown suppressed PC cell growth and invasion^22^. *UCA1* may also be involved in chemoresistance. Several studies have found that high *UCA1* expression correlates with resistance to tamoxifen and cisplatin chemotherapy^23,24^. Li et al. found that silencing of *UCA1* in tamoxifen-resistant breast cancer cell lines increased tamoxifen drug sensitivity by regulating the EZH2/p21 axis and the PI3K/AKT signalling pathway^23^. Additionally, in bladder cancer, high *UCA1* expression is associated with cisplatin resistance^24^. The role of *UCA1* in ENZA resistance in PC has not yet been elucidated. Here, we found that *UCA1* CN gain in cfDNA was independently associated with shorter OS in ENZA-treated as well as in ABI-ENZA-treated mCRPC patients, but not in DOC-treated mCRPC patients. This correlation was even stronger when patients received ABI or ENZA in the first-line setting were considered. Finally, combining *UCA1* with *AR* CN gain analysis of cfDNA substantially improved its predictive value for OS. Based on these findings, *UCA1* is a promising cfDNA serum biomarker for ENZA/ABI resistance in PC.

The other gene selected through exome and transcriptome comparisons was *ORM1*, an acute-phase protein elevated in inflammation, injury, and various cancers^25^. High *ORM1* expression has been linked to tumor grade and poor OS in hepatocellular carcinoma^25^. In PC, Wei et al. analyzed transcriptome data from TCGA and identified seven genes significantly associated with biochemical recurrence, including *ORM1*, where high expression correlated with shorter biochemical recurrence-free survival^26^. The role of *ORM1* in therapy resistance of PC has not yet been evaluated. Our study did not find a significant correlation between *ORM1* CN gain and clinicopathological data or OS in ENZA- and ABI-treated PC patients.

Next-generation antiandrogen therapies are effective treatments for patients with mCRPC; however, baseline or acquired resistance develops frequently. Resistance mechanisms can be categorized as AR-dependent or-independent ones. Several studies showed PC patients carrying *AR* alterations, such as *AR* amplifications (gain), ligand binding-domain point mutations, and splice variants, including *AR-V7* may have distinct outcomes. *AR* gain occurs in 10–15% of plasma samples from treatment-naïve mCRPC patients, compared to 25–50% in those receiving second- or later-line treatments^4^. Moreover, these studies showed that ENZA- and ABI-treated mCRPC patients with *AR* gain had significantly shorter OS and PFS. Furthermore, a meta-analysis revealed that *AR* gain detected in cfDNA was significantly associated with poor survival in ENZA or ABI-treated patients. Conversely, *AR* gain was not associated with OS or PFS in patients who received first-line DOC or second-/third-line cabazitaxel. These data suggest that *AR* gain may indicate that mCRPC patients are more suitable for DOC treatment than for ABI or ENZA treatment^27^. In this study, we analyzed *AR* CN gain in cfDNA samples from three mCRPC cohorts. Our results showed that AR gain was associated with poor OS in the ABI cohort (*p* = 0.039) but not in the ENZA cohort (*p* = 0.990). While this might be attributable to the limited sample size of our cohort, which reduces statistical power and may obscure a true biological effect. Alternatively, this finding may reflect a biological shift in which AR-dependent clones are suppressed under therapy, while resistance is increasingly driven by AR-independent pathways. The PI3K/AKT pathway is a well-established mediator of AR-independent resistance and may serve as a compensatory survival mechanism under ARSI treatment. Accordingly, our in silico analysis revealed that *AKT2* loss co-occurred significantly with *UCA1* gain (*p* = 0.0079, OR = 24.27), whereas AR loss was significantly less frequent in cases with *UCA1* gain (*p* = 8.0 × 10⁻⁵, OR = 0.034). The absence of correlation in the ENZA group may therefore indicate clonal evolution toward an AR-independent phenotype.

Our study has several limitations. As this was a retrospective study, not all clinical data were available. Furthermore, our study cohort had a limited number of cases; the relatively small sample sizes in the ENZA and ABI cohorts may have influenced the stability of the estimated effect sizes, potentially leading to both over- and underestimation of hazard ratios due to limited statistical power and increased susceptibility to random variation. Accordingly, these findings should be interpreted as exploratory, and require validation in larger independent cohorts to confirm their robustness and generalizability. Finally, the functional role of *UCA1* in ENZA resistance has not been investigated; thus, we cannot provide insight into the underlying mechanisms.

On the other hand, our study has notable strengths. First, to our knowledge, this is the first study to identify a biomarker using exome and transcriptome sequencing results from ENZA-sensitive and -resistant PC cell lines. Second, the analyses performed combined experiments at multiple molecular levels, systematically evaluated and validated on clinical samples. Third, we published raw data in the ExAC and GEO databases, which can be used for further studies.

In conclusion, our analysis revealed that *UCA1* gain is associated with poor OS in patients with ENZA - but not DOC-treated mCRPC. Furthermore, *AR* CN gain was associated with reduced OS in ABI-, but not in ENZA- or DOC-treated patients. The combination of *UCA1* and *AR* CN gain substantially increased the predictive value. Patients with *AR* and/or *UCA1* CN gain in cfDNA samples had worse OS than those in the double-negative group. Based on these findings, *UCA1* and *AR* are promising predictive biomarkers for identifying metastatic PC patients with baseline resistance to next-generation antiandrogen treatments.

## Materials and methods

### Cell culturing, exome and transcriptome sequencing, biomarker selection

A detailed description of cell culturing, exome and transcriptome sequencing, as well as biomarker selection, can be found in the Supplementary Materials.

### Patient cohort and samples

Baseline amplification of selected genes was measured in cfDNA samples of 20 patients who underwent ENZA therapy in the first- or later-line mCRPC setting between 04/2012 and 12/2021. As we found pre-treatment *UCA1* CN gain associated with poor survival of ENZA-treated patients, we extended its measurement to cfDNA samples of ABI- and DOC-treated mCRPC patients. The ABI cohort included 20 patients with mCRPC who underwent ABI therapy between 02/2011 and 10/2021. The DOC cohort included 40 patients with mCRPC, who underwent DOC chemotherapy between 09/2017 and 01/2020. The last follow-up was Oct 2023. Samples were collected at the Department of Urology at the Medical University of Vienna and Semmelweis University, Budapest. All patients provided written informed consent to participate in this study prior to enrolment. The study was performed in accordance with ethical standards of the Helsinki Declaration and approved by the ethical boards of the hospitals (Semmelweis University Regional and Institutional Ethics Committee of Science and Research Ethics (TUKEB 55/2014) and Ethics Committee of the University of Vienna (ECS 1986/2017)). PSA response was defined according to the Prostate Cancer Clinical Trials Working Group Criteria (PCWG) II as at least 50% PSA decline from pre-treatment during therapy^28^. OS was selected as the primary endpoint due to its clinical robustness and the limited availability of consistent PSA and radiographic follow-up data in this real-world cohort. OS was calculated as the period between the therapy start and death or censoring. Survival data were retrieved from registry offices.

### cfDNA isolation

Serum samples were collected on the day of the first ENZA, ABI, or DOC treatments. cfDNA was extracted using the Norgen Biotek Cell-Free Circulating DNA Purification Mini Kit (Norgen Biotek, Canada) and quantified using the Qubit™ dsDNA Quantification Assay Kit (Invitrogen, USA). In addition, we used the Agilent TapeStation (Agilent, USA) capillary electrophoresis system for quality assessment.

### Droplet digital PCR

Amplification analysis was performed using a QX200 ddPCR system (Bio-Rad Laboratories). CN assays were performed for *UCA1* (dHsaCNS659969627, FAM, Bio-Rad Laboratories, USA), ORM1 (qHsaCEP0055966, HEX, Bio-Rad Laboratories, USA), and *AR* (dHsaCP2500359, FAM, Bio-Rad Laboratories, USA). Each assay included two negative control samples: one was the non-template control (NTC) sample, and the other was a pooled DNA sample containing DNA from healthy individuals. We used DNA samples isolated from resistant cells as positive controls. Positive and negative clusters were defined using FAM and HEX thresholds, based on the signal amplitudes of the concurrently run positive and negative controls. For each sample, amplification of the selected gene was estimated using the reference gene *ElF2C1* (dHsaCP2500349, HEX, Bio-Rad Laboratories, USA). Absolute CNs at or above two were considered amplifications. PCR reactions were prepared with 5 ng DNA, 10 µl ddPCR Supermix for probes (no dUTP) (Bio-Rad Laboratories, USA) 1 µl primer probe assay and 1 µl EIF2C1 assay in a total volume of 20 µl. Amplification was carried out in a C1000 Touch Thermal Cycler (Bio-Rad Laboratories, USA) using the following ddPCR protocol: 10 min. at 95 °C for enzyme activation, for 30 s at 94 °C for denaturation, and for 1 min. at 60 °C for annealing. After 40 cycles, 98 °C for 10 min. was used for enzyme inactivation. For gene amplification analysis, samples were read on a Bio-Rad QX200 droplet reader using the QuantaSoft software (version 1.7; Bio-Rad Laboratories, USA).

### Statistical analysis

Statistical analysis was performed using SPSS 26.0 (IBM, Chicago, IL, USA) software. Univariate survival analyses were performed using Kaplan-Meier curves with log-rank tests and univariate Cox regression analysis. In all tests, P values < 0.05 were considered statistically significant.

### Gene set enrichment analysis (GSEA)

A detailed description of the GSEA is provided in the Supplementary Materials.

### In silico analysis of *UCA1* gene expression

Publicly available prostate cancer DNA and RNA datasets (https://pmc.ncbi.nlm.nih.gov/articles/PMC4695400/) were analyzed to investigate molecular changes with *UCA1* copy number alterations. Genome-wide CNV analyses were conducted to assess the distribution of copy number gains and losses across genes in relation to *UCA1* status (gain, loss, or neutral). Gene expression analyses were performed to evaluate potential associations between *UCA1* status and selected signaling pathways, including androgen receptor (AR) and PI3K/AKT pathways. Statistical comparisons between groups were performed using Fisher’s exact test. For genome-wide analyses, p-values were adjusted for multiple testing using the Benjamini–Hochberg procedure, with a false discovery rate (FDR) threshold of 5%. For CNV analyses, odds ratios (ORs) and corresponding p-values were calculated. All statistical analyses were conducted using standard bioinformatics tools.

## Supplementary Information

Below is the link to the electronic supplementary material.

Supplementary Material 1

Supplementary Material 2

Supplementary Material 3

Supplementary Material 4

Supplementary Material 5

Supplementary Material 6

Supplementary Material 7

Supplementary Material 8

Supplementary Material 9

Supplementary Material 10

Supplementary Material 11

Supplementary Material 12

Supplementary Material 13

Supplementary Material 14

## Data Availability

The datasets generated and/or analysed during the current study are available in the ENA repository, (PRJEB108829) [[https://www.ebi.ac.uk/ena/browser/view/PRJEB108829](https:/www.ebi.ac.uk/ena/browser/view/PRJEB108829)].
