# Supplementary material for: UCA1 copy number gain in serum cfDNA predicts next-generation antiandrogen resistance in metastatic castration-resistant prostate cancer
Source: Sci Rep. 2026 Jun 21;16:28420. doi: 10.1038/s41598-026-56189-y (PMC13562513; doi:10.1038/s41598-026-56189-y)
Supplement: Supplementary file 1 [file 41598_2026_56189_MOESM14_ESM.docx]

**Supplementary Figure 1:** Gene set enrichment analysis showing normalized enrichment scores (NES) for KEGG pathways. Strong orange bars indicate significant pathways (FDR ≤ 0.05), while light orange and blue bars indicate non-significant ones (FDR > 0.05). Positive NES values reflect enrichment in the ENZA-resistant group; negative values indicate enrichment in the ENZA-sensitive group.

**Table 1.** Patients’ characteristics

**Table 2.** Cox regression analysis of overall survival by clinical and molecular variables in ENZA, ABI, and DOC treatment groups

**Supplementary Table 1**. Association between *AR, UCA1, ORM1* gain and clinicopathological parameters in ENZA-treated patients. * Chi^2^ test was used.

**Supplementary Table 2.** Association between *AR, UCA1, ORM1* gain and clinicopathological parameters in ABI-treated patients. * Chi^2^ test was used.

**Supplementary Table 3.** Association of *AR, UCA1, ORM1* gain with clinicopathological parameters in ENZA&ABI-treated patients. The significant values are indicated in bold. * Chi^2^ test was used.

**Supplementary Table 4.** Association between *AR and UCA1* CN gain and clinicopathological parameters in DOC-treated patients. The significant values are indicated in bold. * Chi^2^ test was used.

**Supplementary Table 5**. Multivariable analysis in patients who underwent ENZA treatment (model 1.)

**Supplementary Table 6**. Multivariable analysis in patients treated ENZA therapy (model 2.)

**Supplementary Table 7**. *In silico* analysis of *UCA1* gain with CNV gain

**Supplementary Table 8**. *In silico* analysis of *UCA1* gain with CNV loss

**Supplementary Table 9**. *In silico* analysis of *UCA1* gain with expression change

**Supplementary Table 10.** Whole exome sequencing data of the ENZA sensitive and resistant cells

**Supplementary Table 11.** Transcriptome sequencing data of the ENZA sensitive and resistant cells
