# Supplementary material for: UCA1 copy number gain in serum cfDNA predicts next-generation antiandrogen resistance in metastatic castration-resistant prostate cancer
Source: Sci Rep. 2026 Jun 21;16:28420. doi: 10.1038/s41598-026-56189-y (PMC13562513; doi:10.1038/s41598-026-56189-y)
Supplement: Supplementary file 2 [file 41598_2026_56189_MOESM1_ESM.docx]

| **Parameters (ENZA)** | **AR gain** | **AR normal** | **p** | **UCA1 gain** | **UCA1 normal** | **p** | **ORM1 gain** | **ORM1 normal** | **p** |
| --- | --- | --- | --- | --- | --- | --- | --- | --- | --- |
| Total Nr. of patients | 9 | 11 |  | 10 | 10 |  | 15 | 5 |  |
| Age | 76 (63 - 83) | 73 (61 - 87) | 0.882 | 76 (68 - 83) | 73 (61 - 87) | 0.436 | 74.6 (63.0 - 87.0) | 72.4 (61.0 - 83.0) | 0.672 |
| PSA (ng/ml) | 178.1 (1.4 - 4341.9) | 21.8 (6.5 - 390.2) | 0.331 | 83.1 (10.6 - 4341.9) | 18.7 (1.4 - 390.2) | 0.089 | 27.0 (1.4 - 886.7) | 66.3 (6.5 - 4342.9) | 0.735 |
| ECOG PS (%) 0 | 3 (16) | 11 (55) | 0.345* | 6 (32) | 8 (40) | 0.510* | 12 (60) | 2 (5) | 0.226* |
| 1 | 5 (26) | 0 (0) |  | 3 (16) | 2 (10) |  | 3 (15) | 2 (5) |  |
| unknown | 1 | 0 |  | 1 | 0 |  | 0 | 1 |  |
| Metastases |  |  |  |  |  |  |  |  |  |
| bone | 8 (40) | 9 (45) | 0.660* | 10 (50) | 7 (35) | 0.060* | 12 (60) | 5 (25) | 0.278* |
| other met.   LN (>2cm) or/and visceral | 4 (20) | 5 (25) | 0.582* | 6 (30) | 3 (15) | 0.361* | 4 (20) | 3 (15) | 0.292* |
| Primary local therapy | 6 (30) | 6 (55) | 0.582* | 5 (25) | 7 (35) | 0.361* | 10 (50) | 2 (10) | 0.292* |
| PSA response (%)>30 | 5 (25) | 8 (40) |  | 5 (25) | 8 (40) |  | 10 (50) | 3 (15) |  |
| >50 | 5 (25) | 7 (35) |  | 4 (20) | 8 (40) |  | 10 (50) | 2 (10) |  |
| >90 | 1 (5) | 7 (35) |  | 3 (15) | 5 (25) |  | 8 (40) | 0 (0) |  |
| Number of patients died | 7 (35) | 7 (35) |  | 8 (40) | 6 (55) |  | 10 (50) | 4 (20) |  |

**Supplementary Table 1**. Association between *AR, UCA1, ORM1* gain and clinicopathological parameters in ENZA-treated patients. * Chi^2^ test was used.
