# Supplementary material for: UCA1 copy number gain in serum cfDNA predicts next-generation antiandrogen resistance in metastatic castration-resistant prostate cancer
Source: Sci Rep. 2026 Jun 21;16:28420. doi: 10.1038/s41598-026-56189-y (PMC13562513; doi:10.1038/s41598-026-56189-y)
Supplement: Supplementary file 3 [file 41598_2026_56189_MOESM2_ESM.docx]

| **Parameters (ENZA&ABI)** | **AR gain** | **AR normal** | **p** | **UCA1 gain** | **UCA1 normal** | **p** | **ORM1 gain** | **ORM1 normal** | **p** |
| --- | --- | --- | --- | --- | --- | --- | --- | --- | --- |
| Total Nr. of patients | 14 | 26 |  | 18 | 22 |  | 29 | 11 |  |
| Age | 74 (63 - 84) | 71 (50 - 87) | 0.440 | 76 (61 - 84) | 71 (50 - 87) | 0.219 | 73 (61 - 87) | 72 (50 - 84) | 0.905 |
| PSA (ng/ml) | 122.5 (1.4 - 4341.9) | 24.4 (0.1 - 941.0) | 0.332 | 83.1 (1.1 - 4341.9) | 17.0 (1.4 - 630.6) | **0.032** | 157.6 (0.1 - 941.0) | 513.7 (6.4 - 4341.9) | 0.550 |
| ECOG PS (%) 0 | 9 (23) | 20 (51) | 0.459* | 12 (31) | 17 (44) | 0.455* | 21 (54) | 8 (21) | 0.750* |
| 1 | 4 (10) | 5 (13) |  | 5 (13) | 4 (10) |  | 7 (18) | 2 (5) |  |
| unknown | 1 | 1 |  | 1 | 1 |  | 1 | 1 |  |
| Metastases |  |  |  |  |  |  |  |  |  |
| bone | 13 (33) | 23 (58) | 0.658* | 17 (43) | 19 (48) | 0.397* | 26 (65) | 10 (25) | 0.906* |
| other met.   LN (>2cm) or/and visceral | 6 (15) | 13 (33) | 0.864* | 12 (30) | 7 (18) | 0.140* | 14 (35) | 5 (13) | 0.522* |
| Primary local therapy | 9 (23) | 11 (28) | 0.158* | 7 (18) | 13 (33) | 0.204* | 16 (40) | 4 (10) | 0.288* |
| PSA response (%) >30 | 7 (18) | 20 (50) |  | 10 (25) | 18 (45) |  | 18 (45) | 9 (23) |  |
| >50 | 6 (15) | 18 (45) |  | 9 (23) | 15 (38) |  | 16 (40) | 8 (20) |  |
| >90 | 1 (3) | 13 (33) |  | 4 (10) | 10 (25) |  | 12 (30) | 2 (5) |  |
| Number of patients died | 10 (71) | 13 (33) |  | 13 (33) | 10 (25) |  | 16 (40) | 7 (18) |  |

**Supplementary Table 3.** Association of *AR, UCA1, ORM1* gain with clinicopathological parameters in ENZA&ABI-treated patients. The significant values are indicated in bold. * Chi^2^ test was used.
