# Supplementary material for: UCA1 copy number gain in serum cfDNA predicts next-generation antiandrogen resistance in metastatic castration-resistant prostate cancer
Source: Sci Rep. 2026 Jun 21;16:28420. doi: 10.1038/s41598-026-56189-y (PMC13562513; doi:10.1038/s41598-026-56189-y)
Supplement: Supplementary file 4 [file 41598_2026_56189_MOESM4_ESM.docx]

| **Parameters (DOC)** | **AR gain** | **AR normal** | **p** | **UCA1 gain** | **UCA1 normal** | **p** |
| --- | --- | --- | --- | --- | --- | --- |
| Total Nr. of patients | 7 | 23 |  | 29 | 11 |  |
| Age | 72 (64 - 82) | 70 (49 - 87) | 0.463 | 71 (49 - 71) | 70 (56 -70) | 0.881 |
| PSA (ng/ml) | 139.8 (54.6 - 6115.4) | 62 (1.0 - 4886.0) | 0.326 | 77.11 (1.0 - 6115.4) | 407.0 (15.3 - 2110.0) | **0.044** |
| ECOG PS (%) 0 | 3 (8) | 14 (35) | 0.520* | 9 (23) | 8 (20) | 0.301* |
| 1 | 4 (10) | 15 (38) |  | 17 (43) | 2 (5) |  |
| 2 | 0 (0) | 4 (10) |  | 3 (8) | 1 (3) |  |
| Metastases |  |  |  |  |  |  |
| bone | 7 (18) | 25 (63) | 0.372* | 22 (55) | 10 (25) | 0.145* |
| other met.   LN (>2cm) or/and visceral | 5 (13) | 15 (38) | 0.288* | 10 (25) | 10 (25) | 0.504* |
| Primary local therapy | 0 (0) | 8 (20) | 0.288* | 2 (5) | 6 (15) | 0.145* |
| PSA response (%) >30 | 3 (8) | 23 (58) |  | 18 (45) | 8 (20) |  |
| >50 | 2 (5) | 18 (45) |  | 14 (35) | 6 (15) |  |
| >90 | 2 (5) | 11 (28) |  | 8 (20) | 5 (13) |  |
| Number of patients died | 5 (13) | 21 (53) |  | 19 (48) | 7 (18) |  |

**Supplementary Table 4.** Association between *AR and UCA1* CN gain and clinicopathological parameters in DOC-treated patients. The significant values are indicated in bold. * Chi^2^ test was used.
