# Supplementary material for: UCA1 copy number gain in serum cfDNA predicts next-generation antiandrogen resistance in metastatic castration-resistant prostate cancer
Source: Sci Rep. 2026 Jun 21;16:28420. doi: 10.1038/s41598-026-56189-y (PMC13562513; doi:10.1038/s41598-026-56189-y)
Supplement: Supplementary file 5 [file 41598_2026_56189_MOESM5_ESM.docx]

| **Parameters (ABI)** | **AR gain** | **AR normal** | **p** | **UCA1 gain** | **UCA1 normal** | **p** | **ORM1 gain** | **ORM1 normal** | **p** |
| --- | --- | --- | --- | --- | --- | --- | --- | --- | --- |
| Total Nr. of patients | 5 | 15 |  | 8 | 12 |  | 14 | 6 |  |
| Age | 73 (70 - 79) | 68 (50 - 84) | 0.349 | 72 (61 - 84) | 69 (50 - 84) | 0.851 | 70.57 (61 - 84) | 71.66 (50 - 84) | 0.444 |
| PSA (ng/ml) | 17.0 (1.7 - 630.6) | 48.2 (0.1 - 941.0) | 0.866 | 89.1 (0.1 - 941.0) | 17.0 (1.7 - 630.6) | 0.305 | 180.6 (0.1 - 941.0) | 53.1 (10.0 - 395.4) | 0.602 |
| ECOG PS (%) 0 | 4 (20) | 11 (79) | 0.946* | 6 (30) | 9 (47) | 0.719* | 9 (47) | 6 (30) | 0.126* |
| 1 | 1 (5) | 3 (21) |  | 2 (10) | 2 (11) |  | 4 (21) | 0 (0) |  |
| unknown | 0 | 1 |  | 0 | 1 |  | 1 | 0 |  |
| Metastases |  |  |  |  |  |  |  |  |  |
| bone | 5 (25) | 14 (70) | 0.554* | 7 (35) | 12 (60) | 0.209* | 14 (7) | 5 (25) | 0.117* |
| other met.   LN (>2cm) or/and visceral | 2 (10) | 8 (40) | 0.787* | 6 (30) | 4 (20) | 0.251* | 5 (25) | 2 (10) | 0.919* |
| Primary local therapy | 3 (15) | 5 (25) | 0.292* | 2 (10) | 6 (30) | 0.264* | 2 (10) | 6 (30) | 0.690* |
| PSA response (%) >30 | 2 (10) | 12 (60) |  | 5 (25) | 9 (45) |  | 8 (40) | 6 (30) |  |
| >50 | 1 (5) | 11 (55) |  | 5 (25) | 7 (35) |  | 6 (30) | 6 (30) |  |
| >90 | 0 (0) | 6 (30) |  | 1 (5) | 5 (25) |  | 4 (20) | 2 (10) |  |
| Number of patients died | 3 (15) | 6 (30) |  | 5 (25) | 4 (20) |  | 6 (30) | 3 (15) |  |

**Supplementary Table 2.** Association between *AR, UCA1, ORM1* gain and clinicopathological parameters in ABI-treated patients. * Chi^2^ test was used.
