# Supplementary material for: UCA1 copy number gain in serum cfDNA predicts next-generation antiandrogen resistance in metastatic castration-resistant prostate cancer
Source: Sci Rep. 2026 Jun 21;16:28420. doi: 10.1038/s41598-026-56189-y (PMC13562513; doi:10.1038/s41598-026-56189-y)
Supplement: Supplementary file 6 [file 41598_2026_56189_MOESM6_ESM.docx]

**Supplemenatry Materials**

**Materials and Methods**

**Cell culture and reagents**

Parental LAPC4, DuCaP, and LNCaP cells were cultured as previously described 1. Therapy-resistant prostate cancer sublines (LAPC4-ER, DuCaP-ER, and LNCaP-ER) were derived as previously described. All the cell lines were incubated at 37°C in a humidified atmosphere. Cell lines were routinely tested for mycoplasma contamination and short tandem repeat analysis was performed to confirm cell line identity.

**Exome sequencing**

DNA samples for exome sequencing and RNA samples for transcriptome analysis were extracted from three biological replicates. Quantity and quality of DNA extracted from cell pellets using the Qiagen AllPrep DNA/RNA Mini Kit (Qiagen, Hilden, Germany) were assessed by Qubit 2.0 Fluorometric Quantitation system (Life Technologies, Carlsbad, CA, USA) and agarose gel electrophoresis, respectively. Libraries were prepared from 100ng input material using the Nextera Flex with Enrichment (currently known as Illumina DNA Prep with Enrichment) kit (Illumina, San Diego, CA, USA) utilizing bead-linked transposomes. Briefly, genomic DNA was tagmented, size-selected and amplified (9 PCR cycles) followed by pooling of individual libraries, hybridization of 500ng pooled PCR products with biotinilated baits and capture with streptavidin-conjugated magnetic beads. After enrichment, library fragments representing in total 45Mb coding region were amplified (7 PCR cycles) and size-selected. Final library pools were quality controlled and sequenced on a HiSeq 4000 instrument (Illumina) using 75bp paired-end chemistry. NGS reads were aligned to the b37 reference genome with BWA MEM (Heng Li, arXiv:1303.3997,https://arxiv.org/abs/1303.3997) before applying GATK (McKenna et al., 2010) base quality score recalibration, indel realignment and duplicate removal. SNP and INDEL discovery as well as genotyping were performed across all samples simultaneously using standard hard filtering parameters or variant quality score recalibration according to GATK Best Practices recommendations (DePristo et al., 2011; Vander Auwera et al., 2013). Somatic variant calling relied on GATK MuTect2.

**Transcriptome sequencing**

**RNA-seq library preparation**

The concentration of total RNA extracted from 3 biological replicates using the Qiagen AllPrep DNA/RNA Mini Kit (Qiagen, Hilden, Germany) was measured with a Qubit 2.0 Fluorometer (Life Technologies, Carlsbad, CA, USA), and the input quality was assessed by determining the RNA integrity number (RIN) using an Experion automated electrophoresis system (Bio-Rad, Hercules, CA, USA). Libraries for transcriptome profiling were prepared with the TruSeq Stranded mRNA LT sample preparation kit (Illumina, San Diego, CA, USA) using Sciclone and Zephyr liquid handling workstations (PerkinElmer, Waltham, MA, USA). Final library concentrations were quantified with the Qubit 2.0 Fluorometer, and the fragment size distribution was assessed using the Experion automated electrophoresis system. Individual libraries were diluted and pooled equimolarly, followed by sequencing on an Illumina HiSeq 4000 instrument using 50bp single-read and 75bp paired-end configurations.

**Raw Data Processing**

After raw data acquisition with HiSeq Control Software v3.4.0.38 (Illumina), base calls provided by the real-time analysis software v2.7.7 (Illumina) were processed with two custom programs based on Picard tools v2.19.2 (<https://broadinstitute.github.io/picard/>). First, base calls were converted into lane-specific multiplexed, unaligned BAM files (IlluminaBasecallsToMultiplexSam, 2.19.2-CeMM) before demultiplexing into sample-specific, unaligned BAM files (IlluminaSamDemux, 2.19.2-CeMM). (Supplementary Table 5)

**Transcriptome Analysis**

Transcriptome analysis was performed with the Tuxedo suite. For each sample, NGS reads passing vendor quality filtering were aligned to the UCSC Genome Browser [hg38] flavour of the Genome Reference Consortium [GRCm38] assembly with TopHat2 (v2.1.1) ^2^, a splice junction mapper utilising the Bowtie2 (v2.2.9) ^3^ short read aligner. Thereby, "basic" Ensembl transcript annotation from version e87 (December 2016) served as reference transcriptome. Cufflinks (v2.2.1) ^4^ allowed for transcriptome assembly, on the basis of spliced read alignments and the reference transcriptome, as well as raw transcript quantification. Before differential expression calling with Cuffdiff (included in Cufflinks v2.2.1) ^5^, transcriptome sets of each sample of each group were combined via the Cuffmerge algorithm. Finally, the cummeRbund([http://www.bioconductor.org/packages/release/bioc/html/cummeRbund.html](https://www.bioconductor.org/packages/release/bioc/html/cummeRbund.html)) and biomaRt ([http://www.bioconductor.org/packages/release/bioc/html/biomaRt.html](https://www.bioconductor.org/packages/release/bioc/html/biomaRt.html)) Bioconductor packages were utilised in custom R scripts to perform quality assessment and further refinement of analysis results.

## [Read Trimming]

NGS reads were trimmed based on quality and adapter sequence content with Trimmomatic in single-end (ILLUMINACLIP:TruSeq3-SE.fa:2:30:10:1:true, SLIDINGWINDOW:4:15, MINLEN:20) or paired-end (ILLUMINACLIP:TruSeq3-PE-2.fa:2:30:10:1:true, SLIDINGWINDOW:4:15, MINLEN:20) mode.

### [STAR Aligner and DESeq2]

NGS reads were mapped to the Genome Reference Consortium [GRCm38] assembly with the “Spliced Transcripts Alignment to a Reference” (STAR) ^6^ aligner utilising the “basic” Ensembl transcript annotation from version [e96] as reference transcriptome. Since the [hg38] assembly flavour of the UCSC Genome Browser was preferred for alignment and downstream data processing with Bioconductor packages for technical reasons, Ensembl transcript annotation had to be adjusted to UCSC Genome Browser sequence region names before the alignment. STAR was run with options suggested by the ENCODE project. Reads overlapping transcript features were counted with the summarizeOverlaps() function of the Bioconductor GenomicAlignments package (<https://bioconductor.org/packages/release/bioc/html/GenomicAlignments.html>), taking into account that the Illumina TruSeq stranded mRNA protocol leads to sequencing of the second strand so that all reads needed inverting before counting. The Bioconductor DESeq2 package (<https://bioconductor.org/packages/release/bioc/html/DESeq2.html>) was then used to model the expression data set and call differentially expressed genes. (Supplementary Table 6)

**Biomarker selection**

To select biomarkers involved in ENZA resistance, we performed comparative whole exome and transcriptome sequencing analysis on three ENZA-resistant and-sensitive prostate cancer cell line pairs. For the comparative analysis, we selected genes that were amplified (as detected by exome sequencing analysis) and had significantly, at least 3-fold, elevated mRNA expression (as detected by RNA-sequencing analysis) in the ENZA-resistant compared to the ENZA-sensitive cell lines.

**Gene set enrichment analysis (GSEA)**

Gene Set Enrichment Analysis (GSEA) was performed to identify biological pathways and molecular processes that were significantly enriched in ENZA-resistant PC cell lines compared to those in ENZA-sensitive ones. GSEA was performed using the WEB-based Gene Set Analysis Toolkit (WebGestalt) (http://www.webgestalt.org/) 18, a comprehensive platform for performing gene set analysis, offering a variety of gene sets from databases such as KEGG (Kyoto Encyclopedia of Genes and Genomes) 19 and Panther and WikiPathways cancer.

**Results**

**Gene Set Enrichment Analysis (GSEA) of ENZA-resistant vs. sensitive Cells**

To identify potentially significant pathways in ENZA resistance, we performed gene set enrichment analysis (GSEA) using the KEGG database. The results revealed distinct enrichment patterns, with pathways significantly enriched in both ENZA-resistant and ENZA-sensitive cells.

Pathways were significantly (FDR ≤ 0.05) downregulated in ENZA-resistant cells (negative Normalized Enrichment Score (NES)) compared to ENZA-sensitive cells, including ribosome-related processes, the coronavirus disease pathway, metabolism of xenobiotics by cytochrome P450, and chemical carcinogenesis. Several immune-related pathways, such as antigen processing and presentation, allograft rejection, and graft-versus-host disease, are also downregulated in resistant cells. These findings suggest that ENZA resistance is associated with decreased immune activity, protein synthesis, and metabolic processing.

In contrast, the pathways upregulated in ENZA-resistant cells (positive NES) included mineral absorption, cell adhesion molecules, neuroactive ligand-receptor interaction, GnRH secretion, motor proteins, Hippo signaling, and cholesterol metabolism. Although these pathways did not reach statistical significance (FDR > 0.05), they indicate potential roles for cell adhesion and GnRH secretion in ENZA resistance (Supplementary Figure 1).

Overall, these results suggest that ENZA resistance is characterized by a reduction in immune-related and protein synthesis pathways, whereas ENZA-sensitive cells exhibit higher expression of metabolic and immune-associated processes.

**References:**

1.	Csizmarik A, Keresztes D, Nagy N, et al. Proteome profiling of enzalutamide-resistant cell lines and serum analysis identified ALCAM as marker of resistance in castration-resistant prostate cancer. *Int J Cancer*. Oct 15 2022;151(8):1405-1419. doi:10.1002/ijc.34159

2.	Kim D, Pertea G, Trapnell C, Pimentel H, Kelley R, Salzberg SL. TopHat2: accurate alignment of transcriptomes in the presence of insertions, deletions and gene fusions. *Genome Biology*. 2013;14(4)doi:10.1186/gb-2013-14-4-r36

3.	Langmead B, Salzberg SL. Fast gapped-read alignment with Bowtie 2. *Nature Methods*. 2012;9(4):357-359. doi:10.1038/nmeth.1923

4.	Trapnell C, Hendrickson DG, Sauvageau M, Goff L, Rinn JL, Pachter L. Differential analysis of gene regulation at transcript resolution with RNA-seq. *Nature Biotechnology*. 2012;31(1):46-53. doi:10.1038/nbt.2450

5.	Trapnell C, Williams BA, Pertea G, et al. Transcript assembly and quantification by RNA-Seq reveals unannotated transcripts and isoform switching during cell differentiation. *Nature Biotechnology*. 2010;28(5):511-515. doi:10.1038/nbt.1621

6.	Dobin A, Davis CA, Schlesinger F, et al. STAR: ultrafast universal RNA-seq aligner. *Bioinformatics*. 2013;29(1):15-21. doi:10.1093/bioinformatics/bts635
